# Evaluation of the therapeutic effect of acoustic therapy combined with acupuncture on idiopathic tinnitus: A randomized controlled trial protocol

**DOI:** 10.1097/MD.0000000000023883

**Published:** 2021-01-22

**Authors:** Siji Wang, Ziqi Chen, Jiaqiu Dai, Fenghui Yu, Houyong Kang

**Affiliations:** The Department of Otorhinolaryngology, The First Affiliated Hospital of Chongqing Medical University, Yuzhong District, Chongqing, China.

**Keywords:** acoustic therapy, acupuncture, idiopathic tinnitus, randomized controlled trial, protocol

## Abstract

**Background:**

There is no effective treatment for idiopathic tinnitus. Both acoustic therapy and acupuncture have been used in the treatment of idiopathic tinnitus, but the clinical efficacy is quite different. For there is no clinical study combining the 2, the purpose of this randomized controlled trial is to evaluate the effectiveness and safety of acoustic therapy combined with acupuncture in the treatment of idiopathic tinnitus.

**Methods:**

This is a prospective randomized controlled trial to study the effectiveness and safety of acoustic therapy combined with acupuncture in the treatment of idiopathic tinnitus, and is approved by the clinical research ethics committee of our hospital. The patients are randomly divided into one of 2 treatment options: (A) acoustic therapy combined with acupuncture group and (B) simple acupuncture group. Patients, doctors, nurses, and data collection assistants are blinded to group allocation. Observation indicators include:

1. Tinnitus Disability Scale;
2. Loudness visual analog scale;
3. Adverse reactions.

Data is analyzed using the statistical software package SPSS version 25.0 (Chicago, IL).

**Discussion:**

This protocol will evaluate the efficacy and safety of acoustic therapy combined with acupuncture in the treatment of idiopathic tinnitus. The results of this experiment will provide clinical evidence for the use of acoustic therapy combined with acupuncture in the treatment of idiopathic tinnitus.

**Trial registration:**

This study protocol is registered in

**Ethics and dissemination:**

Private information from individuals will not be published. This systematic review also does not involve endangering participant rights. Ethical approval was not required.

**OSF Registration number:**

DOI 10.17605/OSF.IO/87VFB.

## 1 Introduction

Tinnitus refers to an abnormal sound consciousness in the ear or head of the patients in the absence of an external sound source.^[1]^ With changes in the social environment, more and more people are exposed to the risk factors of tinnitus, making them susceptible to tinnitus. According to reports, the prevalence of tinnitus is 6.6% to 18.6%, and the prevalence of tinnitus among people over 55 has increased to 30%.^[1,2]^ Tinnitus can be caused by organic, non-organic disease or unknown etiology, and can be divided into objective tinnitus and subjective tinnitus. Unexplained subjective tinnitus is also called idiopathic tinnitus.^[3]^ Due to the high incidence of tinnitus, especially persistent annoying subjective tinnitus can cause anxiety, depression and insomnia in patients, and seriously affect the quality of lives of patients.^[4]^ Some patients even choose to commit suicide because they cannot stand the double torments of severe tinnitus interference and physical and mental illness.^[5]^ Due to the complex etiology of tinnitus and the unclear mechanism, there is still no specific treatment method. Therefore breaking through the dilemma of subjective tinnitus diagnosis and treatment and related research and finding an effective treatment method is still one of the challenges the medical community are facing today.

In recent years, based on the maladaptive auditory cortex reorganization theory in the study of tinnitus mechanism, some researchers have achieved certain results in the application of tailor-made notched music (TMNM) to individualized acoustic therapy for tinnitus patients.^[6]^ Acoustic therapy is a physical and non-invasive treatment that is divided into masking therapy which is similar to tinnitus^[7]^ and a custom therapy that diverts attention, temporarily forgetting or listening to the original sound of nature.^[8]^ The current effective rate reports are quite different. Acupuncture is a traditional treatment method in China, which has a long history of application in the treatment of tinnitus. Studies have shown that acupuncture is effective in the treatment of tinnitus, and the efficacy is due to drug treatment,^[9]^ but there are also systematic reviews indicating that the clinical efficacy of acupuncture in the treatment of tinnitus is not clear yet, the difference is large.^[10,11]^

Therefore, this protocol will combine acoustic therapy and acupuncture in the treatment of idiopathic tinnitus, and evaluate the effectiveness and safety of it.

## 2 Materials and methods

### 2.1 Study design

**Figure 1:**
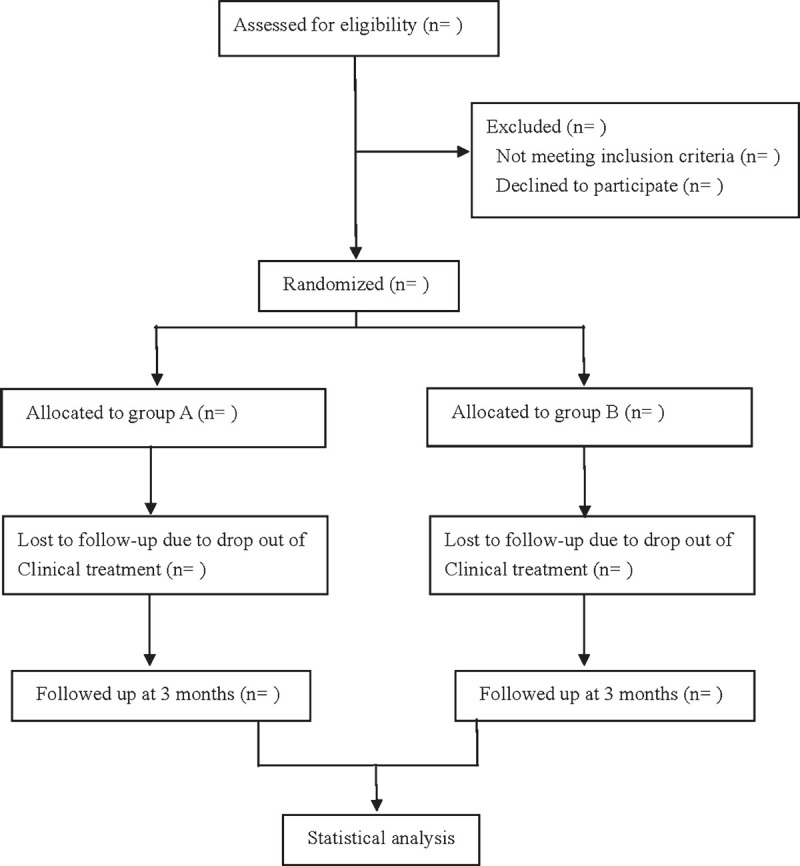
Flow diagram.

This is a prospective randomized controlled trial to study the effectiveness and safety of acoustic therapy combined with acupuncture in the treatment of idiopathic tinnitus. This experiment will follow the intervention reporting standards of acupuncture controlled trials^[12]^ and comprehensive trial reporting standards.^[13]^ The flowchart is shown in Figure 1.

### 2.2 Ethics and registration

This research protocol complies with the Declaration of Helsinki and is approved by the clinical research ethics committee of the hospital. This experiment has already been registered in open science framework (OSF Registration number: DOI 10.17605/OSF.IO/87VFB). Before randomization, all patients need to sign a written informed consent and they are free to choose whether to continue the trial at any time.

### 2.3 Sample size

The calculation of the sample size is based on the visual analogue scale (VAS) score results. According to the pre-test results, the average score of the experimental group is estimated to be 3.1 and the standard deviation is 1.3. The control group average score is 4.2 and the standard deviation is 1.9. The sample size calculation formula is as follows:

$$n=n1=n2=\frac{{\left(u_{a}+u_{\beta }\right)}^{2}\times \sigma^{2}}{\delta^{2}}\times 2$$

At the 5% significance level, a total of 38 patients are required for each group to achieve 80% power. The estimated withdrawal rate is 20%, and 48 patients will be included in each group.

### 2.4 Patients

Inclusion criteria:

1. Meet the diagnosis of idiopathic tinnitus^[3]^;
2. Over 18 years old;
3. Suffering from persistent annoying tinnitus and mainly single tone;
4. The course of the disease is over 6 months and the main frequency of tinnitus is 1000 to 8000 Hz;
5. With or without hearing loss, no severe hearing loss in the octave band centered on the main frequency of tinnitus (≤70 dB HL).

Exclusion criteria:

1. Ear diseases with a clear cause;
2. Objective tinnitus;
3. Pure tone audiometry showed conductive or mixed hearing loss;
4. Suffer from serious systemic diseases (mental and psychological diseases, cervical spondylosis, etc.);
5. Those who cannot understand the research plan after explanation, or are unwilling to participate or have poor compliance.

### 2.5 Study design

Eligible participants are randomly assigned to the treatment group or the control group at a ratio of 1:1 using a random tool based on the central network.^[14]^ Randomization is performed without any stratification and generated by an independent statistician who is not involved in trial implementation or statistical analysis using SAS 9.3 software (SAS Institute, Cary, NC, USA). The clinical research coordinator enter the participant information on the tablet, and is given a random number. The research assistant gets the participants assignment from the computer. Throughout the research process, the research assistant is responsible for screening, recruiting participants, and assigning random number to the included participants. The result assessor is responsible for the assessment of the scale. All of the doctors, researchers, research assistants, participants, intervention supervisors, and statisticians, the grouping of personnel are unknowable.

### 2.6 Intervention before treatment

#### 2.6.1 Listening test

Audiology inspections are all completed in a standard sound insulation room. The inspection items are pure tone hearing threshold, acoustic immittance, and distortion product otoacoustic missions (DPOAE).

#### 2.6.2 Tinnitus test

1. Main tinnitus frequency test: match the patients description of the main tinnitus tone, and obtain the audio that is most similar to the main tinnitus frequency;
2. Tinnitus loudness test: the single-ear sound balance method is used to gradually increase or decrease the intensity of the test tone until it can mask the tinnitus sound, which is the loudness value of the tinnitus matching^[15]^;
3. Based on stability considerations, the selected patients are revised and matched again before start of treatment.

#### 2.6.3 Music processing

The selected music is based on the patient's favorites, 20 familiar music (high-definition, uncompressed, non-defective source code format) is selected from the music network library established by the researcher, and MATLAB coding language is used to perform digital notch filtering on the selected music. Patients with different main frequencies of bilateral tinnitus matching are treated separately for the left and right ears. If the patients tinnitus frequency changes during treatment, it needs to be matched again and the music should be processed accordingly.

### 2.7 Intervention

Therapy group:

1. Music intervention: Distribute customized players with left and right sound channels and over-ear headphones to patients, instruct patients to choose a comfortable loudness and focus on listening in an environment without external sound interference. Continuous or intermittent listening is not less than 2 hours a day for 3 consecutive months.
2. Acupuncture intervention: the patient takes an appropriate position, fully exposes the surgical site, and locally disinfects the acupuncture site (Ermen, Tinggong, Tinghui, Yifeng, Zhongzhu, Xiaxi, Hegu, Taichong. Take the affected side), an acupuncturist with a Chinese medicine practice license and at least 3 years of clinical experience will perform acupuncture operations.

The acupuncture technique is stimulated for 10 seconds to achieve a sense of “deqi”. The needle is retained for 30 minutes, 3 times a week. The course of treatment lasts 3 months.

The control group will be treated with acupuncture alone, and the treatment plan will be the same as that of the treatment group.

### 2.8 Evaluation criteria and efficacy judgment

1. Tinnitus handicap inventory (THI)^[16]^: The THI scale is divided into 5 levels based on the total score. Grade I: Mildly affects life (0–16 points); Grade II: Slightly affects life (18–36 points); Grade III: Moderately affects life (38–56 points); Grade IV: Severely affects life (58–76 points); Grade V: Extremely affects life (78–100 points). The higher the grade, the more serious the degree of tinnitus. Those with a THI score of above 38 are considered moderate and severe tinnitus patients. Efficacy criteria:
  1. Cure: THI score dropped to less than 16 points;
  2. Significantly effective: THI score decreased value ≥17 points;
  3. Invalid: THI score decreased by <17 points, or even increased.^[17]^
2. The visual analogue scale (VAS) marker scale is increased from 0 to 10 points one by one. It is up to the patient to judge the tinnitus loudness and the corresponding tinnitus influence expression map. The higher the score, the greater the tinnitus loudness and its influence. The larger is used to compare the changes in tinnitus loudness values before and after treatment for each patient.
3. Adverse reactions: Including intolerance during treatment, dizziness caused by acupuncture, etc.

### 2.9 Data collection and management

The participants will come to the outpatient clinic for follow-up visits every month for related audiology examinations and tinnitus scale assessments, and 1 or 2 assistants will collect and record the entire data. Personal information about potential participants and registered participants will be collected, shared and stored in an independent storage room to protect confidentiality before, during and after the test.

The access to the database will be restricted to the researchers in this study team.

### 2.10 Statistical analysis

SPSS Version 25.0 (Chicago, IL) is utilized to analyze the age, course of disease, characteristics of tinnitus, THI, VAS score results, and mean value of tinnitus loudness in each group. The measurement data is expressed by $\bar{x}\pm s$. The independent sample *t* test is used for comparing the 2 groups. The Chi-Squared test is used for the composition ratio analysis of tinnitus severity and the difference in curative effect, and the compatibility design data rank sum test and analysis of variance are used for comparison within the group. If *P* is less than .05, the difference will be statistically significant.

## 3 Discussion

Severe tinnitus can affect the patients ability to work, lead to a decrease in the quality of life, and even suicidal tendencies.^[18]^ Its pathogenesis is currently unknown, and may be related to peripheral diseases, central and neuroplasticity, emotional disorders, and the patients mental state. Tinnitus treatment has made great progress in the past 10 years, and there are many existing treatment methods, such as masking, transcranial magnetic stimulation treatment, habituation treatment, medicine, acupuncture, etc., but the effect is not very satisfactory.^[19]^

Acoustic therapy is based on centralization theory. Customized music can increase the release of hypothalamic neurotransmitter dopamine and affect the level of 5-hydroxytryptamine in the brain. By adjusting the level of neurotransmitter in the body, the input pathway can be biased to reduce non-auditory signals. The effect of the joint reduction of the limbic system and parahippocampal gyrus (emotion and memory) on centralizing tinnitus.^[20,21]^ In addition to relying on the traditional meridian theory, the mechanism of acupuncture and moxibustion for the treatment of tinnitus, modern research has confirmed that it is also related to the neurophysiology of the olive cochlear nucleus,^[10]^ the atypical upregulation subcortical auditory pathway, the limbic system and the amygdala,^[22]^ and the plasticity of the nervous system^[23,24]^ and so on. The 2 treatments have been applied in clinical practice, but the clinical efficacy is not satisfactory, so we try to combine the 2 treatments to treat specific tinnitus.

This study also has the following limitations: Because this is a single-center randomized controlled study, the included population is regionalized, and the results may be biased; Due to the treatment method, this study cannot achieve strict double-blind and the observation indicators are highly subjective, which may have certain impact on the results.

## Author contributions

**Conceptualization:** Siji Wang, Ziqi Chen.

**Data curation:** Siji Wang, Ziqi Chen.

**Formal analysis:** Jiaqiu Dai, Fenghui Yu.

**Funding acquisition:** Houyong Kang.

**Software:** Siji Wang, Ziqi Chen.

**Supervision:** Houyong Kang.

**Writing – original draft:** Siji Wang, Ziqi Chen.

**Writing – review & editing:** Siji Wang, Houyong Kang.
